# Biostimulants induce positive changes in the radish morpho-physiology and yield

**DOI:** 10.3389/fpls.2022.950393

**Published:** 2022-08-08

**Authors:** Qurat-Ul-Ain Raza, Muhammad Amjad Bashir, Abdur Rehim, Rafia Ejaz, Hafiz Muhammad Ali Raza, Umbreen Shahzad, Faraz Ahmed, Yucong Geng

**Affiliations:** ^1^Department of Soil Science, Faculty of Agricultural Sciences and Technology, Bahauddin Zakariya University, Multan, Pakistan; ^2^College of Agriculture, Bahauddin Zakariya University Multan, Bahadur Sub-Campus Layyah, Layyah, Pakistan; ^3^Soil and Water Testing Laboratory, Sargodha, Pakistan; ^4^KOYO Star Agriculture Technology Co., LTD., Beijing, China

**Keywords:** biostimulants, fertilization, glycine, radish, vegetable production

## Abstract

An ever-increasing population has issued an open challenge to the agricultural sector to provide enough food in a sustainable manner. The upsurge in chemical fertilizers to enhance food production had resulted in environmental problems. The objective of the current study is to assess the utilization of biostimulants for sustainable agricultural production as an alternative to chemical fertilization. For this purpose, two pot experiments were conducted to examine the response of radish against individual and combined applications of biostimulants. In the first experiment, the effects of chemical fertilizer (CK), glycine (G), lysine (L), aspartic acid (A), and vitamin B complex (V) were studied. The results demonstrated that V significantly improved the transpiration rate (81.79%), stomatal conductance (179.17%), fresh weight (478.31%), and moisture content (2.50%). In the second experiment, tested treatments included chemical fertilizer (CK), Isabion^®^ (I), glycine + lysine + aspartic acid (GLA), moringa leaf extract + GLA (M1), 25% NPK + M1 (M2). The doses of biostimulants were 5g L^−1^ glycine, 1g L^−1^ lysine, 2g L^−1^ aspartic acid, and 10 ml L^−1^ moringa leaf extract. The photosynthetic rate improved significantly with GLA (327.01%), M1 (219.60%), and M2 (22.16%), while the transpiration rate was enhanced with GLA (53.14%) and M2 (17.86%) compared to the Ck. In addition, M1 increased the stomatal conductance (54.84%), internal CO_2_ concentration (0.83%), plant fresh weight (201.81%), and dry weight (101.46%) as compared to CK. This study concludes that biostimulants can effectively contribute to the sustainable cultivation of radish with better growth and yield.

## Introduction

The main challenge the world is facing today is to feed its increasing population using less and highly efficient inputs (Bashir et al., 2021). It is estimated that by 2050 the population will increase to 9.7 billion; this demands a 70% increase in food production to feed such a huge population (Del Buono, 2021). On the other hand, the key factors influencing crop productivity are limited available land, climate change, the genetic potential of crops, and depletion of natural resources (Maja and Ayano, 2021). The Green Revolution was a promising approach to food security, but in the long run, its adverse impact on agriculture, human health, and the environment were observed worldwide (John and Babu, 2021).

There is rampant overuse of chemical fertilizers and about 1.9 × 10^11^ tons of them are used globally (Wan et al., 2021). Conventional agricultural patterns that include excessive use of fertilizers and pesticides have increased crop production but have negatively impacted food quality and environmental sustainability (Chouhan et al., 2021). The excessive use of chemical fertilizers contributes to groundwater and atmospheric pollution, poor food quality, increased input cost with lower outputs, deterioration of the soil quality and fertility status, reduced nutrient efficiency, susceptibility to pathogens, nutrient leaching, greenhouse gases emission, and other abiotic stresses (Rahman and Zhang, 2018; Godlewska et al., 2021; Wan et al., 2021).

To improve crop yield, quality, and environmental sustainability, various breeding programs have been attempted. The need for sustainable and modernized agriculture makes it imperative to improve plant growth and development functions, stimulate plant metabolism, and improve environmental safety (Bashir et al., 2021). Biostimulants are organic/ inorganic substances gaining popularity due to their incredible benefits for better plant growth and development. They are involved in the stimulation of plant physiological processes and improved plant shoot/ root growth. They minimize chemical fertilizer use, are cost-effective, enhance plant's tolerance to biotic and abiotic stresses, boost yield, and, more importantly, offer an environmentally friendly approach (Bashir et al., 2021; Rehim et al., 2021; Bell et al., 2022; Samuels et al., 2022). European Biostimulant Industry Council (EBIC) reported that biostimulants improve the nutrient use efficiency and uptake in plants, counteract the influence of biotic and abiotic stresses, and improve crop quality (Bashir et al., 2021). Based on their origin, biostimulants are generally classified as humic, amino-acid/nitrogen-containing, inorganic, chitosan/ biopolymers, beneficial microbes, seaweed, and botanical extracts (Samuels et al., 2022).

One of the beneficial effects of biostimulants to be identified recently is crop yield enhancement. Moringa leaf extract is a potent bio-stimulator that improves plant growth and yield. It contains essential nutrients, sugars, amino acids, phytohormones, vitamins, and antioxidants. It benefits the plants by improving seed germination, nutrient status, growth and yield processes, fruit quality, chlorophyll content, and biometric attributes (Abd El-Mageed et al., 2017; Arif et al., 2022). Moreover, using biostimulants also promotes nutrient uptake, enzymatic activity, crop physiology, green leaf pigment, plant metabolism, and molecular processes (Rehim et al., 2021).

The use of amino acid-based biostimulants positively impacts the plants' metabolic processes and favors sustainable agriculture (Alfosea-Simón et al., 2020). In addition, animal-based protein hydrolysates significantly increase the gaseous exchange and chlorophyll fluorescence (Cristiano and De Lucia, 2021). Without chemical fertilizers, the foliar application of biostimulants, vitamins, and coenzyme Q10 significantly improves the radish root-shoot biomass under green-house conditions (Rehim et al., 2021). Radish is of various cultivars, including shapes (oval, round, spindle, icicle, cylindrical, long, conical, half long), color (white, red, pink, black, purple), and flavor. The crop is cultivated worldwide on a large scale; its extensive cultivation is better due to its taste, high nutritional content, low calories, and myriad health benefits (Godlewska et al., 2021). In Asia, it is one of the most economically important crops; it is cultivated for its soft leaves and root, which is consumed raw, dried, pickled, or simmered (Lee and Park, 2020). Radish has also been reported to reduce health risks associated with cancer, stone formation, constipation, jaundice, and anxiety (Godlewska et al., 2021). As per published data, biostimulants play a vital role in the yield enhancement of various vegetables along with controlled chemical fertilization. Still, there is a considerable gap in identifying the potential benefits of biostimulants for morpho-physiological parameters of radish. This makes the current study timely and important to identify the influence of biostimulants on sustainable agriculture.

Considering the positive effects of biostimulants on plant growth, development, and yield, the present study was undertaken with the following objectives: (i) identify the physiological response of radish against individual and combined use of biostimulants, (ii) estimate the efficacy of biostimulants on crop morphology, and (iii) find an alternative to minimize the use of chemical fertilizers. We hypothesized that the use of biostimulants will not just only improve the radish production but will also enhance the photosynthetic rate and other related physiological indices.

## Materials and methods

Two pot experiments were conducted to assess the role of biostimulants on radish yield, morphology, and physiology. A brief description of each experiment is given below.

### Experiment 1

The experiment was conducted from September to December 2021 at the research area of the Department of Soil Science (30.258° E, 71.515° N), Bahauddin Zakariya University, Multan, Pakistan. The climate of the Multan region is semi-arid to arid. The pots were lined with plastic sheets and filled with 15 kg soil having the following physicochemical properties: pH 7.7, EC 0.5 dS·m^−1^, organic matter 0.4%, saturation percentage 34%, texture loam, nitrogen (N) 221 mg kg^−1^, phosphorus (P) 8.2 mg kg^−1^, and potassium (K) 30.1 mg kg^−1^.

The experiment consisted of five treatments with three replications in a completely randomized design (CRD). The tested treatments were: recommended chemical fertilizer (CK; N 61.8 kg ha^−1^, P 49.4 kg ha^−1^, K 61.7 kg ha^−1^), glycine (G; C_2_H_5_NO_2_), lysine (L; C_6_H_14_N_2_O_2_), aspartic acid (A; C_4_H_7_NO_4_), and vitamin B complex (V; vitamin B1, B6, B12). The doses of biostimulants were maintained the same according to the crop's N requirements (61.8 kg ha−1). G, L, and A were purchased from Sigma Aldrich, while V was purchased from Martin Dow Market Ltd.

Six radish seeds (cultivar “Mino early long white,” Nongwoo Bio) were sown in each pot on 24 September 2021. Smaller seedlings were later eradicated, and only two plants per pot were maintained. The foliar application was performed four times in the morning on sunny and windless days (15 October, 25 October, 5 November, and 15 November). Plants were harvested on 9 December 2021. Regular manual weed eradiation and irrigation practices were maintained during the growth period. Plant samples (rosette and root) were collected after harvesting for further analysis.

### Experiment 2

The experiment was conducted between October 2021 and January 2022 at the research area of the College of Agriculture (30.97° E, 70.96° N), Bahauddin Zakariya University, Bahadur Sub-campus Layyah, Pakistan. The region has a desert climate with usually no rainfall, and similar to experiment one, the pots were lined with plastics sheets and filled with 8 kg soil (pH 7.8, EC 0.1 dS·m^−1^, organic matter 0.7%, saturation percentage 28%, texture sandy loam, N 450 mg kg^−1^, P 7.1 mg kg^−1^, and K 62.34 mg kg^−1^).

The experiment comprised five treatments with three replications in a completely randomized design (CRD). The treatments included: recommended chemical fertilizer (CK; N 61.8 kg ha^−1^, P 49.4 kg ha^−1^, K 61.7 kg ha^−1^), Isabion^®^ (I), glycine + lysine + aspartic acid (GLA), moringa leaf extract + GLA (M1), 25% NPK + M1 (M2). The doses of biostimulants were 5 g L^−1^ glycine, 1 g L^−1^ lysine, 2 g L^−1^ aspartic acid, and 10 ml L^−1^ moringa leaf extract. Whereas, Isabion^®^ was used as recommended by the manufacturer. G, L, and A were purchased from Sigma Aldrich, while I was purchased from Syngenta, Pakistan. Moringa leaf extract was prepared at the laboratory. Fresh moringa (50 g) leaves were weighed and soaked in 200 ml distilled water for 12 h, then sonicated, centrifuged, and stored supernatant for further use.

Similar to experiment one, six seeds were sown in each pot on 21 October 2021, and two healthy plants were maintained. Four foliar applications of biostimulants were applied on 16 November, 26 November, 6 December, and 16 December 2021. The crop was harvested on 13 January 2022, and plant rosette and root samples were collected for further analysis.

### Gaseous exchange parameters

Gaseous exchange parameters (photosynthetic rate, transpiration rate, internal CO_2_, and stomatal conductance) were measured at vegetative maturity (ten days before harvesting) using an infrared gas analyzer (IRGA; Analytical Development Company, Hoddesdon, UK). Readings were taken between 8:00 and 9:00 a.m. The IRGA chamber was attached to a fresh expanded radish leaf and logged for ten seconds (Scafaro et al., 2018; Zaheer et al., 2020).

### Photosynthetic pigments

To determine the chlorophyll a + b content, fresh leaf samples (0.5 g) were collected and cut into fine pieces (<0.25 mm^2^). The samples were dipped in ethanol (96% v/v) for chlorophyll extraction and placed in darkness for 24 h. Afterward, the spectrophotometer was used to determine the chlorophyll a + b contents by measuring absorbance at 665 and 649 nm. The following equation was used to calculate the chlorophyll a + b contents (mg g^−1^).


$$\begin{array}{l} \text{Chlorophyll a }+\text{ b }=\frac{\left(5.1\times \text{ A}665+\;20.24\;\times \text{ A}649\right)\;\times \text{V}}{\text{m}} \end{array}$$

Where A665 and A649 is the absorbance value, V is the volume of solution (liters), and m is the weight (g) of the sample used (Yuan et al., 2016).

### Leaf greenness index

The leaf greenness index was measured using SPAD 502 Plus Chlorophyll Meter (Konica Minolta, Osaka, Japan). Three fully expanded fresh leaves from each pot were selected, and the readings were measured three times. The average was calculated to estimate leaf greenness.

### Fresh and dry weight

Plant samples (rosette and roots) were collected and washed at harvest, and fresh plant weight was measured. Later on, the samples (root and rosette) were oven-dried at 65°C until a constant weight was achieved. The plant dry weight was measured using a weighing balance and recorded in grams.

### Moisture content

The moisture content in samples was determined using the following formula.


$$\begin{array}{l} \;Moisture\;content\;(\%)=\\ \frac{(\text{Fresh weight}-\;\text{Oven dry weight})\;}{\text{Fresh weight}}\;\times \;100 \end{array}$$

Where fresh weight is the weight of plant samples at the time of harvest, and oven-dry weight was obtained after drying the samples at 65°C until a constant weight was achieved. A weighing balance was used for weighing, and readings were taken.

### Biometric analysis

The biometric analysis of radish included root diameter and length. After harvest, the radish was washed, and its length was measured using a measuring tape. Whereas, the diameter of radish was determined using Vernier calipers.

### Statistical analysis

Data sets were represented as means ± standard deviation (SD), and CRD was the basis of the analysis of variance (ANOVA). The least significant difference (LSD) test was used for comparison at a 5% probability level. Statistical softwares R-studio^®^ and Statistix 9^®^ were used for statistical analysis. Microsoft Excel 2016 was used for data processing and visualization. Pearson correlation analysis was performed using R-studio.

## Results

The radish growth performance improved significantly with the use of biostimulants. In our experiments, the control group contained plants sprayed with the recommended dose of chemical fertilizers (CK). All the treatments are compared with CK.

### Experiment 1

#### Gaseous exchange parameters

Photosynthetic rates showed insignificant changes with the identified values as 4.03 μmol m^−2^ s^−1^ in CK, 4.23 μmol m^−2^ s^−1^ in G, 4.00 μmol m^−2^ s^−1^ in L, 4.61 μmol m^−2^ s^−1^ in A, and 3.78 μmol m^−2^ s^−1^ in V respectively. Transpiration rate was significantly increased with the application of V (81.79%), followed by A (47.77%), L (36.77%), and G (1.17%), respectively, compared with CK. The identified values were 0.97 mmol m^−2^ s^−1^ in CK, 1.17 mmol m^−2^ s^−1^ in G, 1.33 mmol m^−2^ s^−1^ in L, 1.43 mmol m^−2^ s^−1^ in A, and 1.76 mmol m^−2^ s^−1^ in V respectively. Stomatal conductance was also increased with a similar trend where V showed the highest increase (179.17%), followed by A (141.67%), L (87.50%), and G (47.92%). The values observed were 0.16 mmol m^−2^ s^−1^ in CK, 0.24 mmol m^−2^ s^−1^ in G, 0.30 mmol m^−2^ s^−1^ in L, 0.39 in A, and 0.45 mmol m^−2^ s^−1^ in V. Internal CO_2_ concentration was significantly improved with the application of biostimulants. The highest increase was observed with G (15.04%), L (14.69%), V (13.81%), and A (11.70%), respectively, compared with CK. The values reported were 379.00 μmol mol^−1^ in CK, 436.00 μmol mol^−1^ in G, 434.67 μmol mol^−1^ in L, 423.33 μmol mol^−1^ in A, and 431.33 in V.

#### Photosynthetic pigments

**Figure 1:**
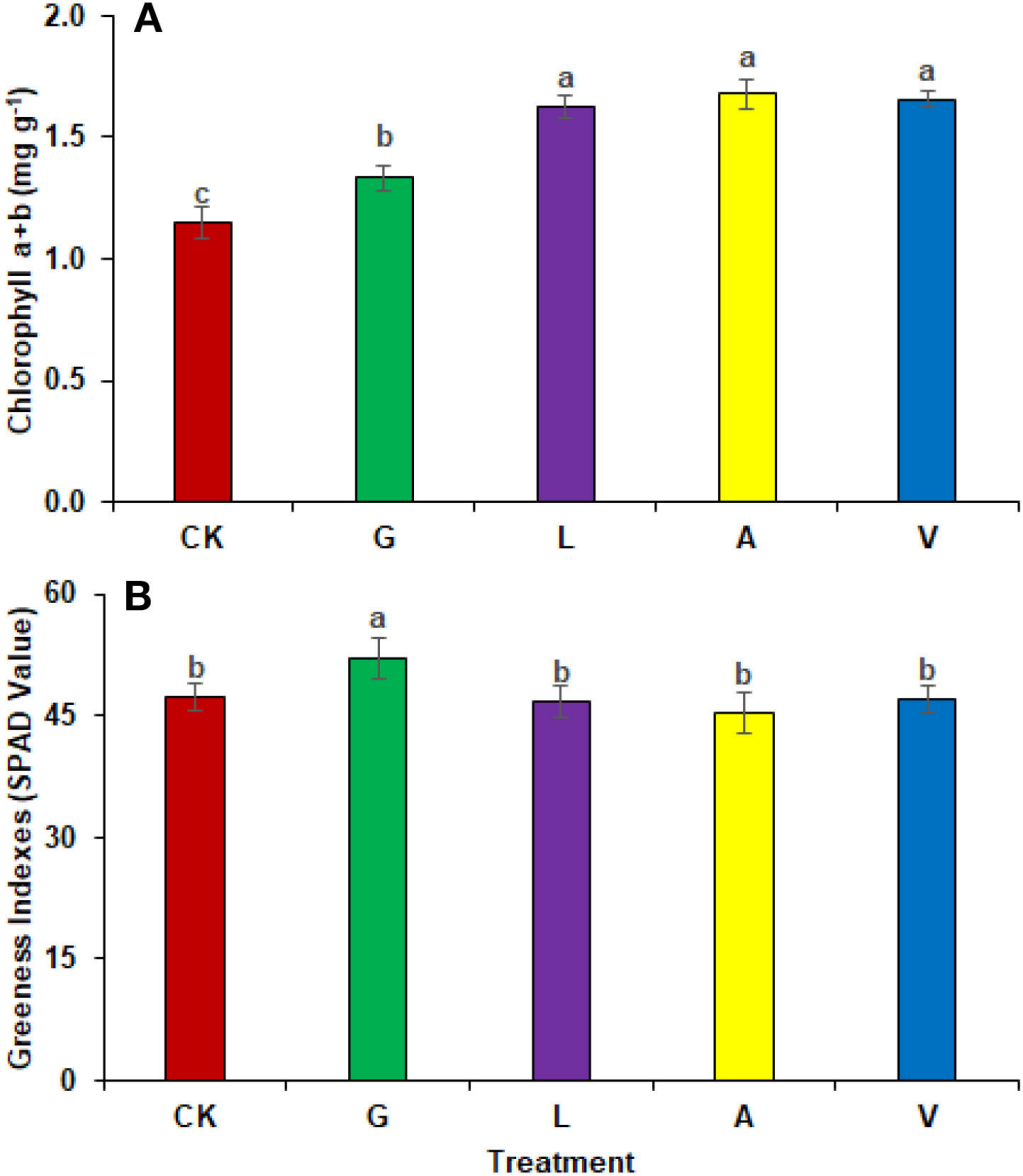
Pigment analysis: **(A)** Photosynthetic pigments **(B)** Leaf greenness indexes observed in experiment 1 using chemical fertilizer (CK), glycine (G), lysine (L), aspartic acid (A), and vitamin B complex (V). The values mentioned herein are indicated as mean ± S.D and the lowercase letters indicate the significant difference among the means.

The foliar application of biostimulants on radish increased the photosynthetic pigments in leaves. Chlorophyll contents were improved significantly with the application of A (45.81%), followed by V (43.89%), L (41.28%), and G (16.01%) as compared to CK (Figure 1A). The identified values were 1.15 mg g^−1^ in CK, 1.33 mg g^−1^ in G, 1.62 mg g^−1^ in L, 1.68 mg g^−1^ in A, and 1.65 mg g^−1^ in V, respectively. The SPAD measurements demonstrated that G enhanced (9.91%) the greenness index of the leaves, while the values observed were 47.31 in CK, 52.00 in G, 46.70 in L, 45.37 in A, and 46.99 in V (Figure 1B).

#### Fresh and dry weight

**Figure 2:**
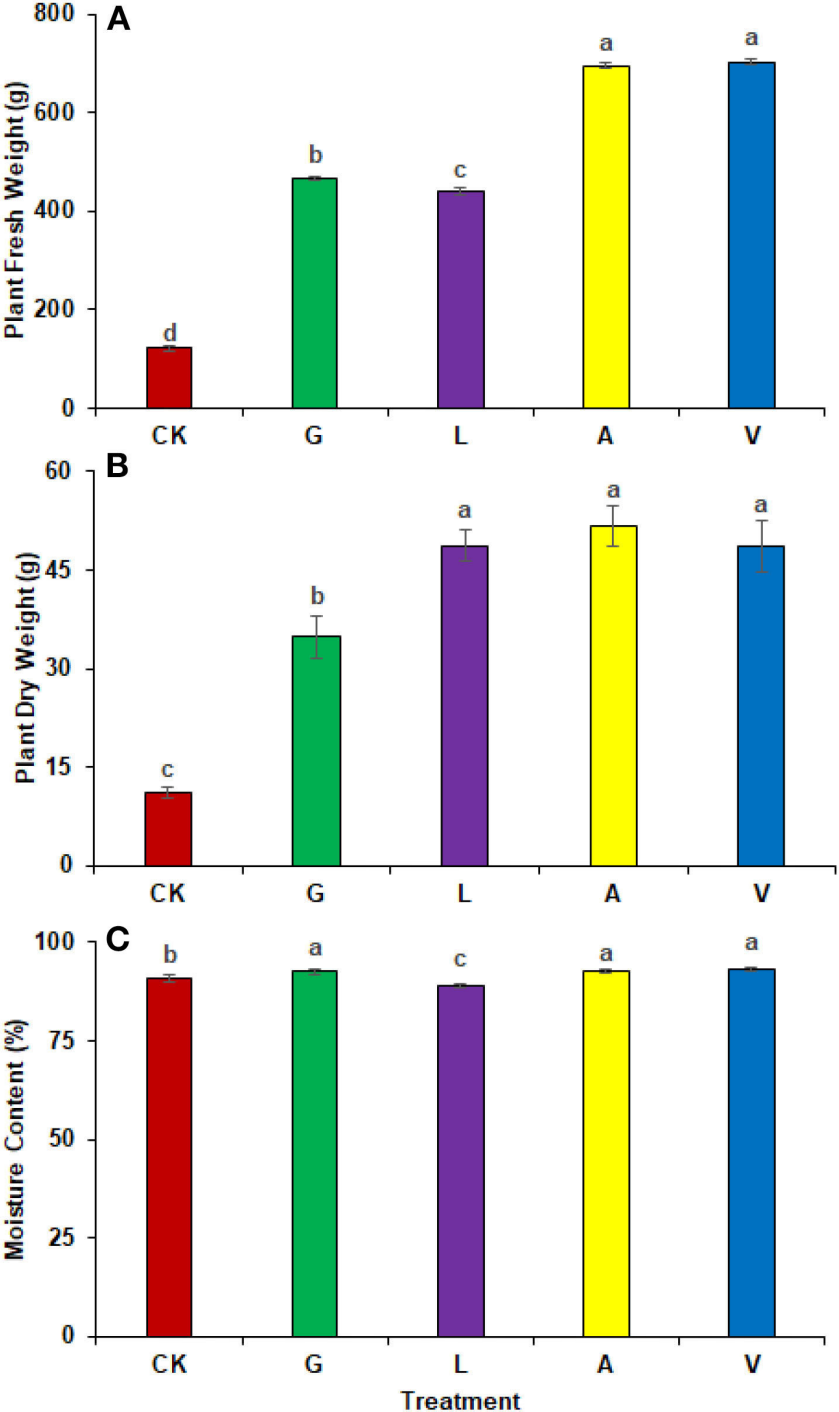
Plant weight and moisture content: **(A)** Plant fresh weight, **(B)** Plant dry weight, **(C)** Moisture content observed in experiment 1 using chemical fertilizer (CK), glycine (G), lysine (L), aspartic acid (A), and vitamin B complex (V). The values mentioned herein are indicated as mean ± S.D and the lowercase letters indicate the significant difference among the means.

A significant improvement in plant total fresh and dry weights were observed with the application of biostimulants. The identified values were 121.33 g in CK, 466.67 g in G, 441.67 g in L, 695.00 g in A, and 701.67 g in V, respectively. The most significant change in fresh plant weight was observed in radish plants receiving V (478.31%), followed by A (472.82%), G (284.63%), and L (264.02%) (Figure 2A). In addition, plant dry weight improved significantly in A, followed by L, V, and G (364.73, 337.90, 364.73, and 212.50%) compared to the CK (Figure 2B). The identified values were 11.33 g in CK, 34.78 g in G, 48.74 g in L, 51.72 g in A, and 48.61 g in V.

#### Moisture content

The use of biostimulants influenced radish moisture contents. The moisture contents were higher in V (2.50%), followed by A (1.94%), and G (1.92%) as compared to CK. Whereas, the identified values were 90.80% in CK, 92.55% in G, 88.97% in L, 92.56% in A, and 93.07% in V respectively (Figure 2C).

#### Morphological analysis

**Figure 3:**
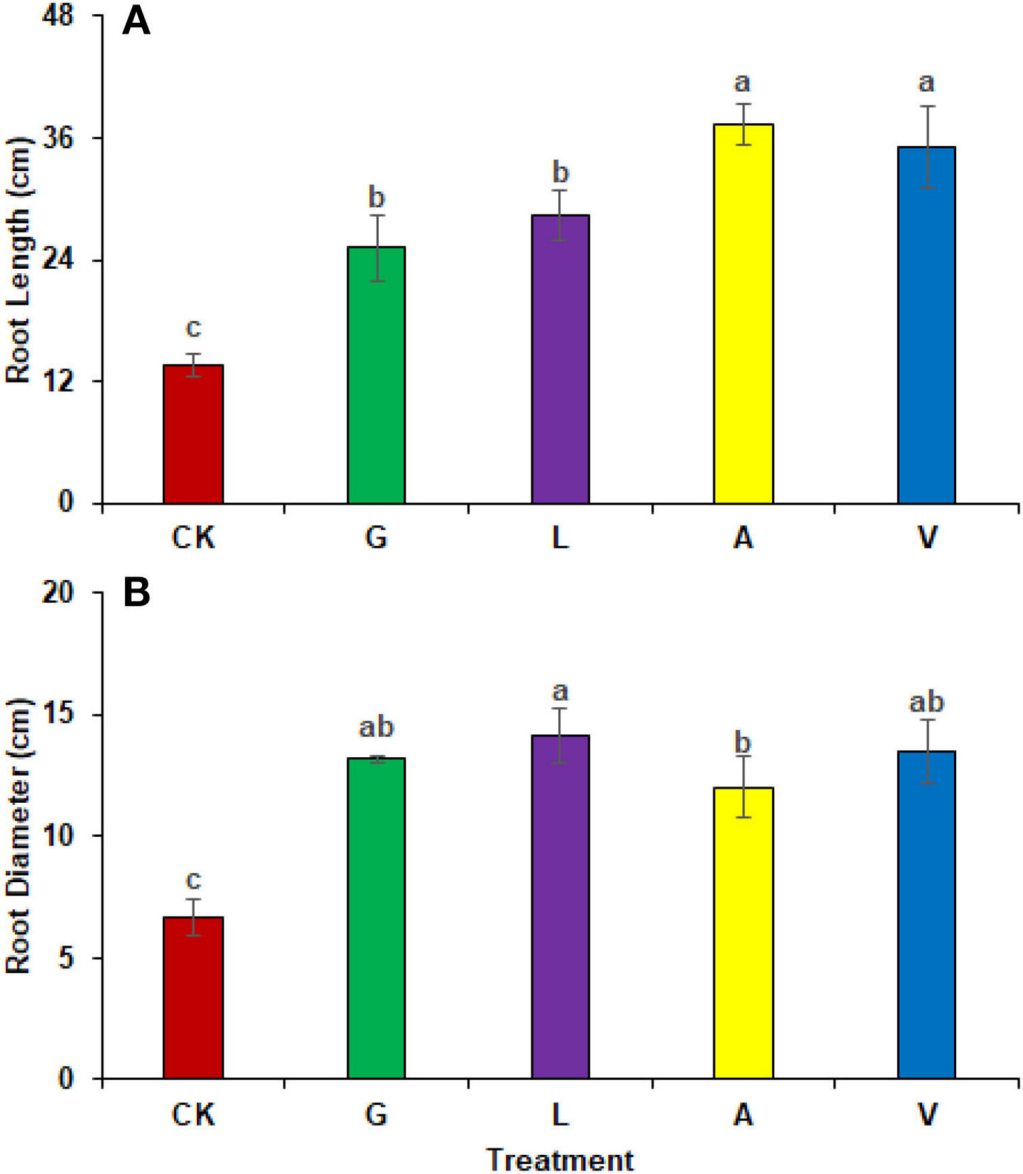
Biometric attributes of radish: **(A)** Root length **(B)** Root diameter observed in experiment 1 using chemical fertilizer (CK), glycine (G), lysine (L), aspartic acid (A), and vitamin B complex (V). The values mentioned herein are indicated as mean ± S.D and the lowercase letters indicate the significant difference among the means.

The biostimulants influenced the root length and diameter significantly. Root length was increased significantly with A (172.86%), followed by V (157.50%), L (107.27%), and G (84.10%) in comparison with CK. The identified values were 13.67 cm in CK, 25.17 cm in G, 28.33 cm in L, 37.30 cm in A, and 35.20 cm in V, respectively (Figure 3A). Similarly, root diameter was increased with L (111.89%), V (102.40%), G (97.40%), and A (79.91%) as compared to CK. The values observed were 6.67 cm in CK, 13.17 cm in G, 14.13 cm in L, 12.0 cm in A, and 13.50 cm in V, respectively (Figure 3B).

### Experiment 2

#### Gaseous exchange parameters

**Figure 4:**
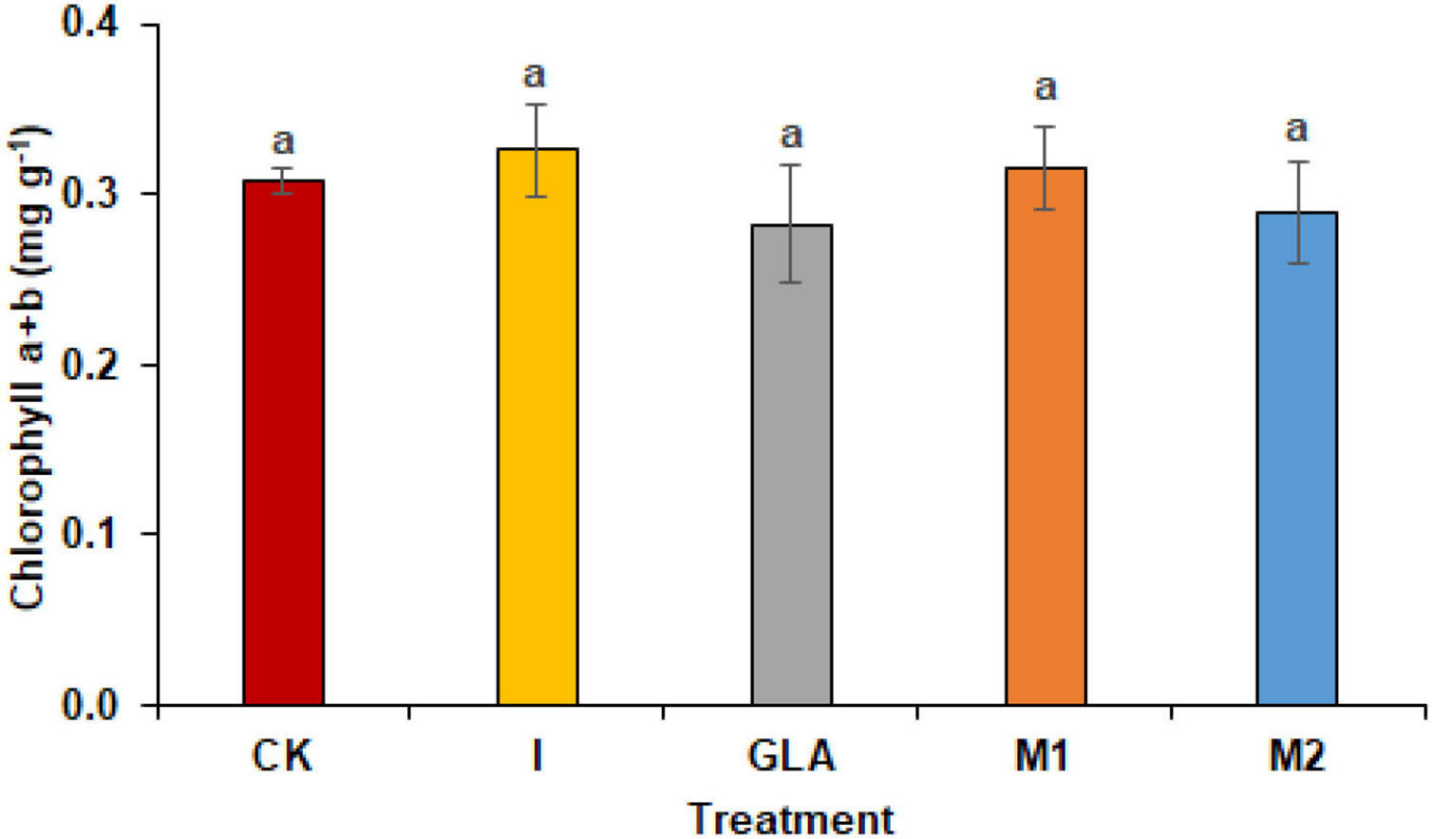
Photosynthetic pigments observed in experiment 2 using chemical fertilizer (CK), Isabion^®^ (I), glycine + lysine + aspartic acid (GLA), moringa leaf extract + GLA (M1), 25% NPK + M1 (M2). The values mentioned herein are indicated as mean ± S.D and the lowercase letters indicate the significant difference among the means.

Similar to experiment one, biostimulants significantly improved the plants' physiology in experiment two (Figure 4). Photosynthetic rates were significantly enhanced with GLA (327.01%), M1 (219.60%), and M2 (22.16%), respectively. The identified values were 3.64 μmol m^−2^ s^−1^ in CK, 3.42 μmol m^−2^ s^−1^ in I, 15.54 μmol m^−2^ s^−1^ in GLA, 11.63 μmol m^−2^ s^−1^ in M1, and 4.45 μmol m^−2^ s^−1^ in M2 respectively. The transpiration rate was significantly improved with GLA (53.14%) and M2 (17.86%) compared to the CK. The identified values were 4.46 mmol m^−2^ s^−1^ in CK, 4.31 mmol m^−2^ s^−1^ in I, 6.83 mmol m^−2^ s^−1^ in GLA, 4.03 mmol m^−2^ s^−1^ in M1, and 5.26 mmol m^−2^ s^−1^ in M2 respectively. In addition, the stomatal conductance was enhanced significantly with M1 (54.84%) followed by GLA (43.01%), and internal CO_2_ concentration was improved with M1 and M2 (0.83 and 1.93%, respectively) compared to CK. Whereas, the values of stomatal conductance were 0.31 mmol m^−2^ s^−1^ in CK, 0.30 mmol m^−2^ s^−1^ in I, 0.44 mmol m^−2^ s^−1^ in GLA, 0.48 mmol m^−2^ s^−1^ in M1, and 0.28 mmol m^−2^ s^−1^ in M2 respectively. Moreover, the values of internal CO_2_ concentration were 363.33 μmol mol^−1^ in CK, 344.67 μmol mol^−1^ in I, 338.67 μmol mol^−1^ in GLA, 366.33 μmol mol^−1^ in M1, and 370.33 in M2.

#### Photosynthetic pigments

The photosynthetic pigments of radish leaves showed non-significant results with the spraying of biostimulants. Chlorophyll a + b was highest in I (5.11%) and M1 (1.47%) as compared to CK. The identified values were 0.31 mg g^−1^ in CK, 0.33 mg g^−1^ in I, 0.28 mg g^−1^ in GLA, 0.31 mg g^−1^ in M1, and 0.29 mg g^−1^ in M2 respectively (Figure 4).

#### Fresh and dry weight

**Figure 5:**
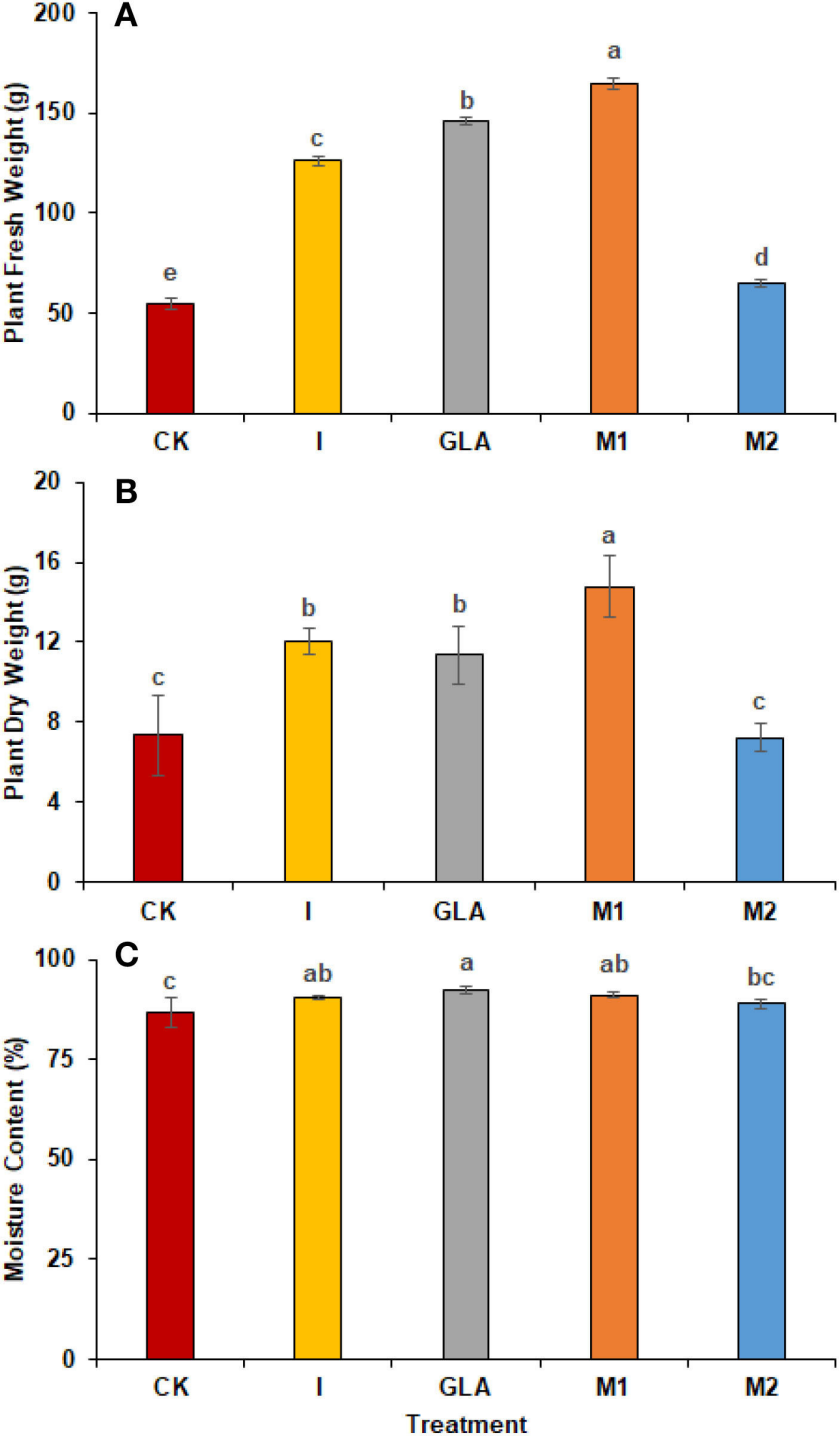
Plant weight and moisture content: **(A)** Plant fresh weight, **(B)** Plant dry weight, **(C)** Moisture content observed in experiment 2 using chemical fertilizer (CK), Isabion^®^ (I), glycine + lysine + aspartic acid (GLA), moringa leaf extract + GLA (M1), 25% NPK + M1 (M2). The values mentioned herein are indicated as mean ± S.D and the lowercase letters indicate the significant difference among the means.

A significant increase in plant total fresh and dry weights were observed with the application of biostimulants. The highest plant fresh weight was achieved in plants receiving M1 (201.81%), followed by GLA (167.06%), I (130.47%), and M2 (18.29%). The identified values were 54.67 g in CK, 126.00 g in I, 146.00 g in GLA, 164.00 g in M1, and 64.67 g in M2, respectively (Figures 5A,B). In addition, plant dry weight was significantly improved in M1, followed by I and GLA (101.46%, 63.84%, and 54.93% higher than CK). The observed values were 7.33 g in CK, 12.01 g in I, 11.36 g in GLA, 14.77 g in M1, and 7.22 g in M2 (Figure 5C).

#### Moisture content

**Figure 6:**
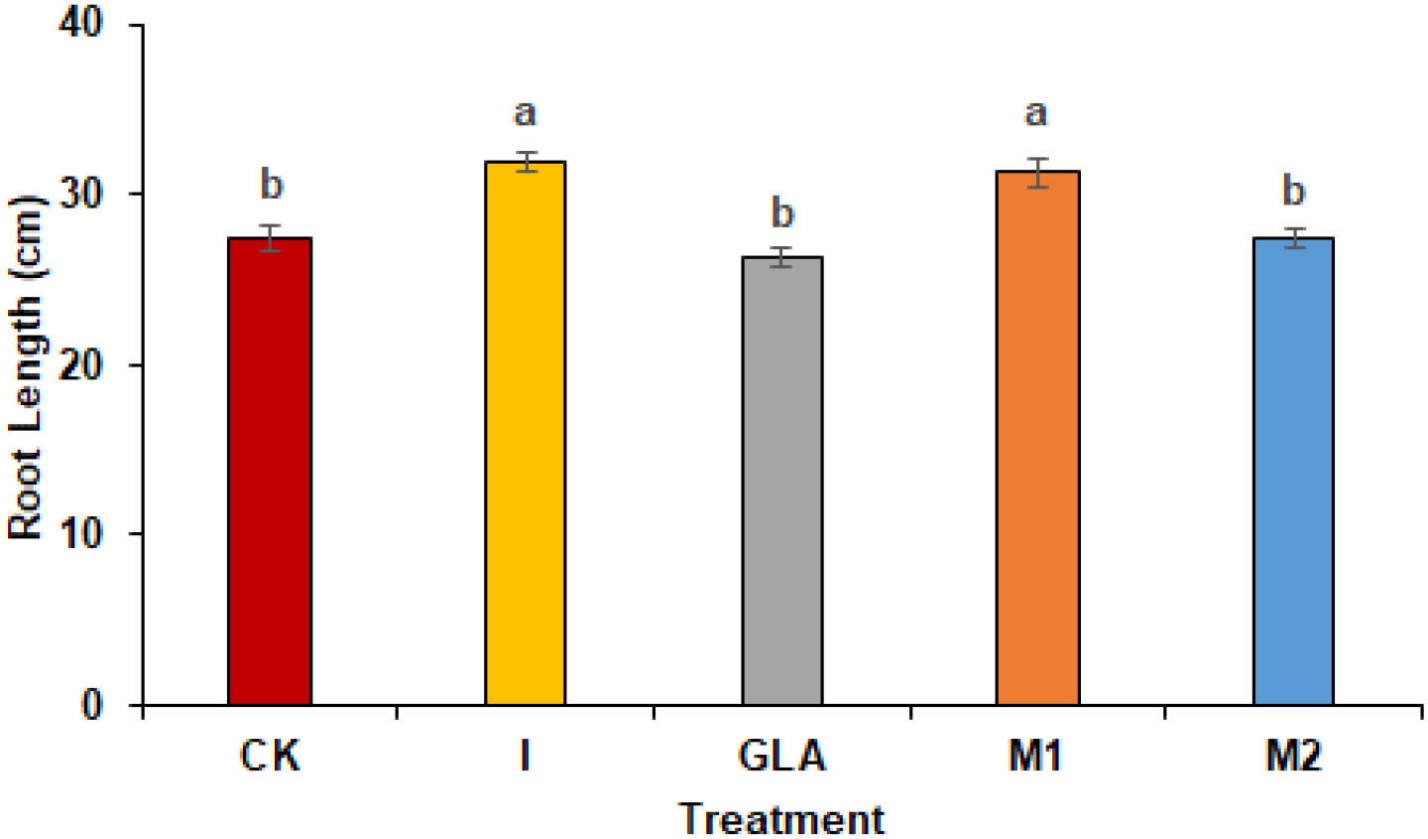
Root length of radish observed in experiment 2 using chemical fertilizer (CK), Isabion^®^ (I), glycine + lysine + aspartic acid (GLA), moringa leaf extract + GLA (M1), 25% NPK + M1 (M2). The values mentioned herein are indicated as mean ± S.D and the lowercase letters indicate the significant difference among the means.

Moisture contents were also influenced by the use of biostimulants and were significantly improved with GLA (6.51%) followed by M1 (5.16%) and I (4.48%) as compared to CK. The identified values were 86.59% in CK, 90.47% in I, 92.23% in GLA, 91.06% in M1, and 88.82% in M2 respectively (Figure 6).

#### Morphological analysis

**Figure 7:**
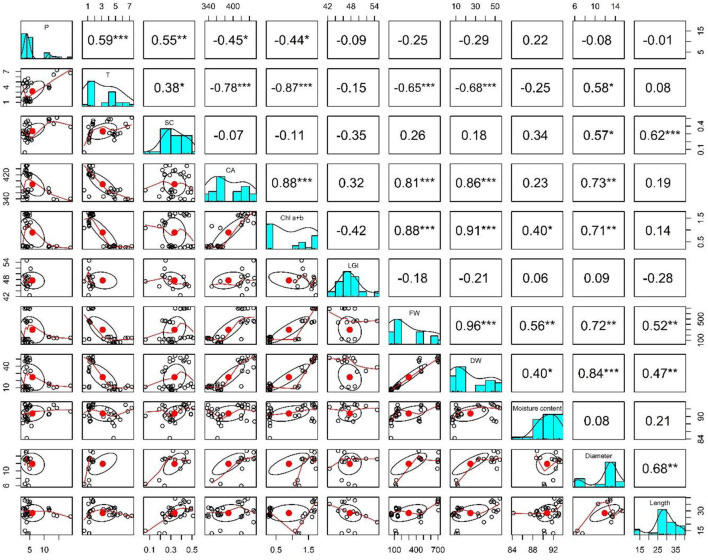
Pearson correlation indicating the relation between assessment parameters: P is Photosynthetic rate, T is Transpiration rate, SC is Stomatal conductance, CA Internal CO_2_ concentration, Chl a+b is Photosynthetic pigments, LGI is Leaf greenness indexes, FW is Plant fresh weight, DW is Plant dry weight, Moisture content is Moisture content, Diameter is Root diameter, and Length is Root length. The * indicates week relationship, ** shows strong, and *** shows very strong relationship.

Radish length was influenced significantly by the use of biostimulants and was shown by I (16.52%) followed by M1 (14.09%) and M2 (0.35%) as compared to CK. The values observed were 27.42 cm in CK, 31.91 cm in I, 26.33 cm in GLA, 31.28 cm in M1, and 27.52 cm in M2, respectively (Figure 7).

#### Pearson correlation

Photosynthesis is strongly positively correlated with transpiration rate and moderately associated with stomatal conductance. It has a weak negative correlation with internal CO_2_ assimilation and chlorophyll content. Transpiration showed a strong negative association with internal CO_2_ assimilation, chlorophyll content, fresh weight, and dry weight. Moreover, a weak positive correlation was observed between stomatal conductance and root diameter. Stomatal conductance showed a strong positive association with root length and a weak positive relationship with root diameter. Internal CO_2_ concentration strongly correlates with chlorophyll content, fresh weight, and dry weight. In addition, a moderate positive relation with root diameter was observed. Chlorophyll content showed a strong positive correlation with fresh and dry weight, moderate relation with root diameter, and a weak association with moisture content. Plant fresh weight had a strong positive relation with dry weight and moderate association with moisture content, root length, and diameter. Moreover, dry weight had a strong positive correlation with root diameter, moderate correlation with root length, and a weak association with moisture content. Root length and diameter were also positively correlated with each other (Figure 7).

## Discussion

An increasing population along with climate change has threatened agricultural production. Today's need is to develop new sustainable products to increase production and yield and support organic farming (Godlewska et al., 2021). In this scenario, biostimulants can play an environment-friendly role to increase crop productivity, which is associated with the minimal use of chemical fertilizers. In the current study, individual and the combined application of biostimulants showed a potential to improve radish morphology, growth, and yield.

**Figure 8:**
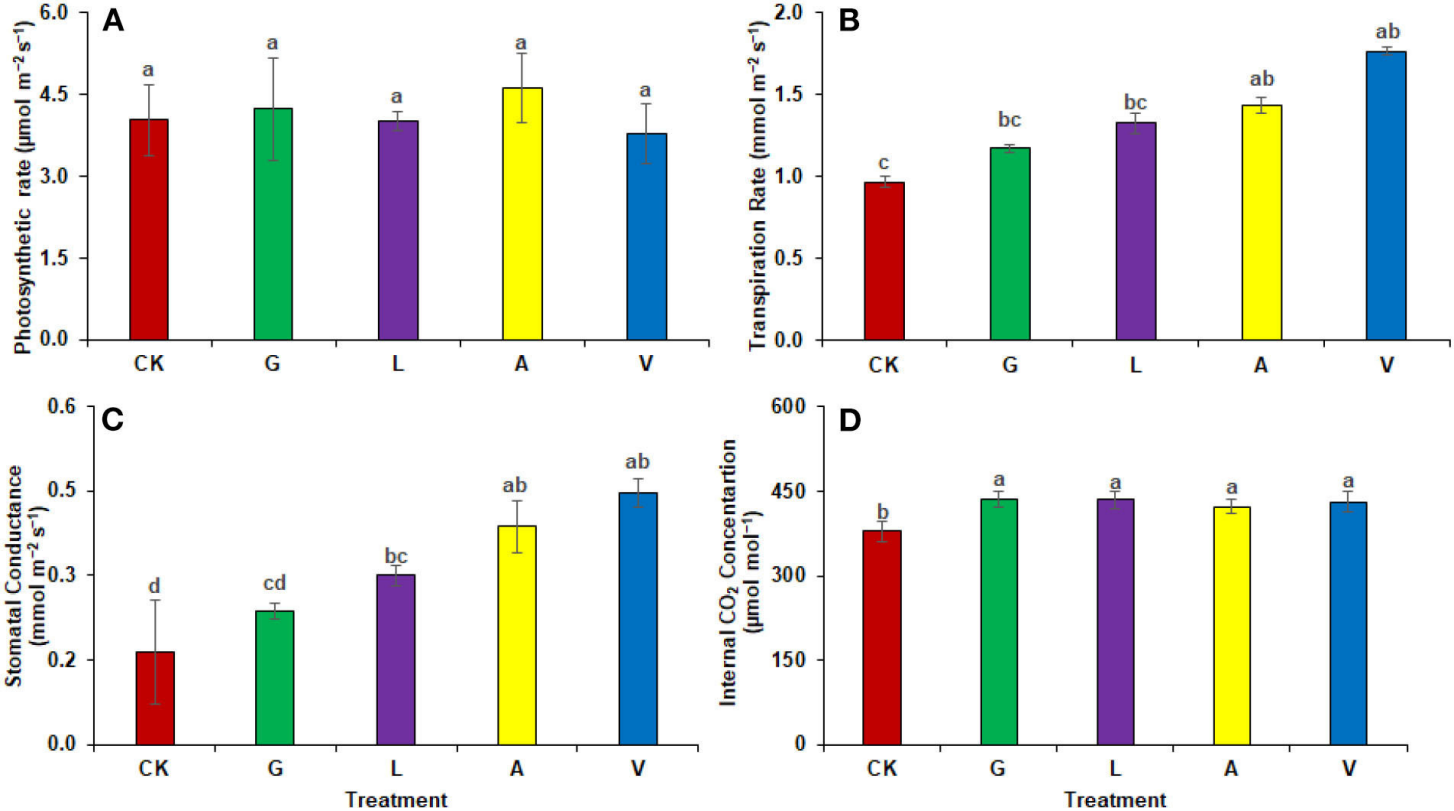
Gaseous exchange parameters: **(A)** Photosynthetic rate, **(B)** Transpiration rate, **(C)** Stomatal conductance **(D)** Internal CO_2_ concentration observed in experiment 1 using chemical fertilizer (CK), glycine (G), lysine (L), aspartic acid (A), and vitamin B complex (V). The values mentioned herein are indicated as mean ± S.D and the lowercase letters indicate the significant difference among the means.

**Figure 9:**
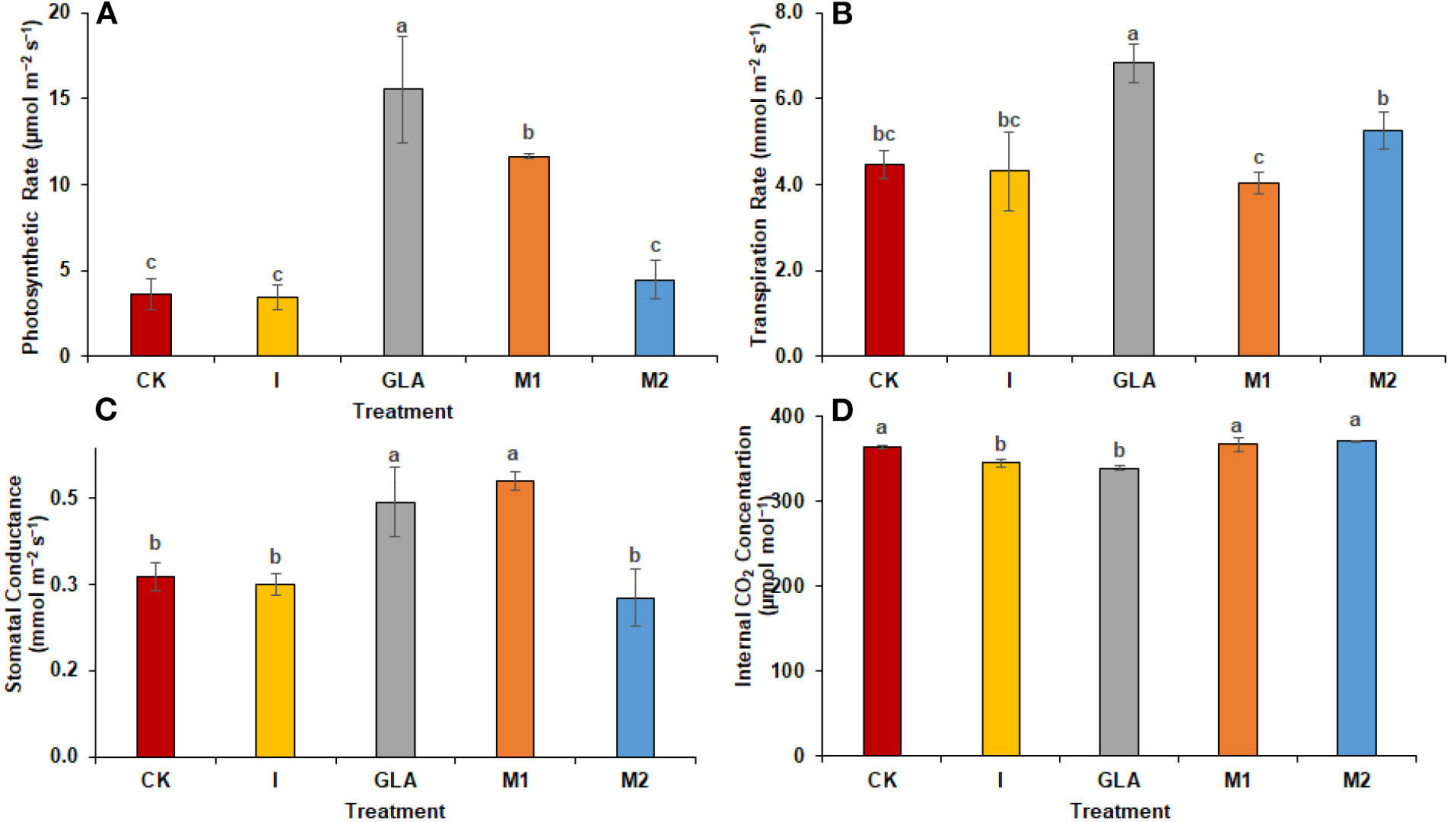
Gaseous exchange parameters: **(A)** Photosynthetic rate, **(B)** Transpiration rate, **(C)** Stomatal conductance **(D)** Internal CO_2_ concentration observed in experiment 2 using chemical fertilizer (CK), Isabion^®^ (I), glycine + lysine + aspartic acid (GLA), moringa leaf extract + GLA (M1), 25% NPK + M1 (M2). The values mentioned herein are indicated as mean ± S.D and the lowercase letters indicate the significant difference among the means.

Our results demonstrated an increase in leaf gas exchange attributes. The use of biostimulants improved the transpiration rate, stomatal conductance, and internal CO_2_ in plants (Figures 8, 9). Biostimulants can potentially improve water-use efficiency in plants (Jiménez-Arias et al., 2022), which also increases turgor pressure in radish leaf guard cells and increases gaseous exchange attributes. Leaf greenness and chlorophyll are essential in transmitting and absorbing solar energy (Ahmad et al., 2021). The results showed that amino acid-based biostimulants significantly improved the chlorophyll content (Figures 1, 4). The increased pigments with biostimulants can be associated with water and ion use efficiency, better stomatal conductance, photosynthetic capacity, and bioactive compounds, i.e., vitamins (Ali et al., 2020), amino acids, and mineral nutrients (Bahmani Jafarlou et al., 2022). In addition, moringa leaf extract also improves chlorophyll content with its induced sink capability through the supply and translocation of photo-assimilates from leaves to other parts of a plant that improve fruit quality (Arif et al., 2022).

In addition, the use of biostimulants showed a positive response to radish yield and growth. The results determined that the use of A, V, and M1 significantly improved the radish yield and production (Figures 2, 5). Whereas a low yield was observed with the combined use of biostimulants and mineral fertilizers. The amino acid-based biostimulants positively impact plant physiology and metabolism, while the interaction of biostimulants when applied in combination is still unknown (Alfosea-Simón et al., 2021). Plants can produce amino acids and vitamins, but it requires considerable energy; therefore, if applied exogenously, they can save energy and improve their development at different growth stages (Popko et al., 2018). In addition, improved yield can also be related to the potential of Vitamins B_1_ and B_6_ to develop resistance in plants against oxidative stress (Asensi-Fabado and Munné-Bosch, 2010). Biostimulants also act as signal-transducing molecules and benefit plant physiological processes that contribute to higher yield and biomass (Khan et al., 2019).

The results of both experiments also suggest that foliar application of amino acids and vitamins improves the radish root morphology (Figures 3, 6). Previous literature reported that root growth is also stimulated with the application of biostimulants having vitamin B-complex (Rehim et al., 2021). Vitamins B_1_ is a cofactor that contributes significantly to improve primary metabolic processes in plants (glycolysis, tricarboxylic acid cycle, and pentose phosphate pathway) that ultimately increases plant growth performances (Soppelsa et al., 2019). In addition, free amino acids are small molecules easily absorbed by plant leaves and roots and promote endogenous biosynthesis of plant hormones that ultimately regulate plant phenological processes (Caruso et al., 2019). Foliar application of amino acids improves plant growth, yield, and morphology in beans and radish (Kocira et al., 2020; Rehim et al., 2021). Amino acids are involved in synthesizing organic compounds and phytohormones (auxin and gibberellin) and enhancing macro and micronutrient uptake, contributing to better crop productivity (Kocira et al., 2020). Moreover, glycine has a better potential to release N molecules than urea (McCoy et al., 2020).

Although the use of chemical fertilizers contributes a lot to improving crop productivity and yield, it has adverse effects on the soil, water, and environment. The results of our study demonstrated that the foliar application of biostimulants has a promising role in improving crop yield, physiology, and morphology. Moreover, it is an innovative method for the sustainable cultivation of radish. However, the long-term impact and effects of biostimulants on soil properties and field conditions need to be explored.

## Conclusion

The demand for food and a safe environment is gaining attention from the scientific community, and sustainable agriculture needs effective chemical fertilizers, genetically improved plants, and growth-promoting biostimulants. Biostimulants are a rich source of biologically active compounds and can potentially improve plant metabolic processes to achieve better crop quality, yield, and productivity. The study concludes that the foliar application of biostimulants increases gaseous exchange activities and chlorophyll content in radish as well as the plant's morphology, and its fresh and dry biomass, which can be conclusive evidence to reduce chemical fertilizers. However, further studies regarding field conditions, long-term uses and impacts, effects on soil mineral status, nutrient use efficiency, and plant biochemical properties are the identified research gaps. In addition, the mechanistic approaches behind the improved results with the combined use of biostimulants need attention.

## Data availability statement

The original contributions presented in the study are included in the article/supplementary material, further inquiries can be directed to the corresponding authors.

## Author contributions

All authors listed have made a substantial, direct, and intellectual contribution to the work and approved it for publication.

## Funding

This study acknowledges the financial aid provided by Higher Education Commission Pakistan under the indigenous Ph.D. 5000 fellowship program 520(PH-II) 2AV6-075/HEC/IS/2020 for Q-U-AR to pursue her Ph.D. at BZU Multan.

## Conflict of interest

Author YG was employed by KOYO Star Agriculture Technology Co., LTD. The remaining authors declare that the research was conducted in the absence of any commercial or financial relationships that could be construed as a potential conflict of interest.

## Publisher's note

All claims expressed in this article are solely those of the authors and do not necessarily represent those of their affiliated organizations, or those of the publisher, the editors and the reviewers. Any product that may be evaluated in this article, or claim that may be made by its manufacturer, is not guaranteed or endorsed by the publisher.
